# Age-dependent expression changes of circadian system-related genes reveal a potentially conserved link to aging

**DOI:** 10.18632/aging.203788

**Published:** 2021-12-19

**Authors:** Emanuel Barth, Akash Srivastava, Diane Wengerodt, Milan Stojiljkovic, Hubertus Axer, Otto W. Witte, Alexandra Kretz, Manja Marz

**Affiliations:** 1 Bioinformatics/High Throughput Analysis, Faculty of Mathematics and Computer Science, Friedrich Schiller University Jena, Jena, Germany; 2 FLI Leibniz Institute for Age Research, Jena, Germany; 3 Hans Berger Department of Neurology, Jena University Hospital, Jena, Germany; 4 German Center for Integrative Biodiversity Research (iDiv), Halle-Jena-Leipzig, Germany; 5 European Virus Bioinformatics Center (EVBC), Jena, Germany

**Keywords:** aging, circadian clock system, circadian rhythm, inter-species comparison, longevity, RNA-Seq

## Abstract

The circadian clock system influences the biology of life by establishing circadian rhythms in organisms, tissues, and cells, thus regulating essential biological processes based on the day/night cycle. Circadian rhythms change over a lifetime due to maturation and aging, and disturbances in the control of the circadian system are associated with several age-related pathologies.

However, the impact of chronobiology and the circadian system on healthy organ and tissue aging remains largely unknown. Whether aging-related changes of the circadian system’s regulation follow a conserved pattern across different species and tissues, hence representing a common driving force of aging, is unclear.

Based on a cross-sectional transcriptome analysis covering 329 RNA-Seq libraries, we provide indications that the circadian system is subjected to aging-related gene alterations shared between evolutionarily distinct species, such as *Homo sapiens*, *Mus musculus*, *Danio rerio*, and *Nothobranchius furzeri*. We discovered differentially expressed genes by comparing tissue-specific transcriptional profiles of mature, aged, and old-age individuals and report on six genes (*per2*, *dec2*, *cirp*, *klf10*, *nfil3*, and *dbp*) of the circadian system, which show conserved aging-related expression patterns in four organs of the species examined. Our results illustrate how the circadian system and aging might influence each other in various tissues over a long lifespan and conceptually complement previous studies tracking short-term diurnal and nocturnal gene expression oscillations.

## INTRODUCTION

The life of most organisms is, beyond other influences, controlled by two central biological events: Biological rhythms based on the timely regulation of physiological processes, and the biology of aging. Circadian rhythms (CRs) are established by a genetically encoded circadian program, controlling a multitude of biological cycles and enabling an individual to adjust to periodic environmental changes during daytime, seasons, and lifetime. Thus, its conserved nature allows to synchronize metabolic, endocrine, behavioral, and complex intracellular events across a 24-hour day/night cycle (for a recent review, see [1]).

Aging is an equally complex process, which is affected by a plethora of exogenous and endogenous factors, and which impacts virtually all crucial biological processes by a progressive loss of cellular functions [2, 3]. It is well established that aging interferes with the regulation of the circadian system, which, in return, contributes to the manifestation and progression of aging-related diseases (reviewed in [4, 5]). Furthermore, age-independent alterations of the circadian system can result in premature aging of vertebrates and invertebrates, suggesting a direct mutual interrelation between both processes [6].

As superordinate pacemaker of the circadian system in mammals, the suprachiasmatic nucleus (SCN) of the anterior hypothalamus imposes CRs on gene expression in each tissue and cell type. Thus, it conveys rhythmicity to all essential processes of physiology and behavior. In parallel with whole-body circadian rhythmicity imprinted by the SCN, circadian oscillations are regulated autonomously at the organ and cellular levels, with a functional clock residing in peripheral tissues and cell types. In comparison with mammals, including humans, CRs in lower vertebrates, such as fish, are less hierarchically organized and do not depend on the SCN [7]. Accordingly, the entrainment of CRs via environmental and endogenous cues is more potent in fish, where organs such as the skin and liver directly respond to light exposure and hormonal and nutritive conditions.

The functionality of the CRs extenuates with aging, generally manifesting in a lower penetrance of their rhythmicity. This decline is characterized by reduced amplitudes and increased scatter in circadian acrophases, disturbances of cell and tissue synchronization, and phase shifts in oscillations over a 24-hour cycle [8–10]. Likewise, fragmentation and shifts in the periodicity of CRs are prominent in elder mice and humans [9, 10], where they influence rest-activity and sleep-wake period patterns. In addition to causing sleep disorders, disturbances in CRs are associated with pronounced stress responses, impaired DNA repair, and cancer. They are considered an independent risk factor for age-related disorders such as type II diabetes mellitus, Alzheimer’s dementia, coronary heart disease, and tumors [11–14].

Furthermore, CR misalignment is associated with a loss in protein homeostasis, which, in turn, represents a common feature of various age-related proteinopathies, particularly those manifesting in the nervous system [15]. Accordingly, genes with a role in the surveillance and orchestration of CR signatures are essential in the control of biological processes at the individual cell and complex network levels, as they influence cellular maintenance, chromatin integrity and DNA repair, epigenetic modifications, autophagy, metabolic homeostasis, and immune functions. All these functions are fundamentally involved in the processes of cellular senescence and tissue aging [2]. Recent analyses in mammals reported that at least 40% of the protein-coding genes are subjected to circadian oscillations, and those genes correlated with age-related dysfunctions are intimately connected to CR drivers [11].

However, it is not well studied in how far changes in the expression of CR core genes and their downstream targets are responsible for age-related variations in CR regulation. Moreover, it is unclear to what extent these changes in CR gene regulatory patterns may overlap in distinct tissues and species. A related open question remains as to whether a conserved age-related pattern of CR gene expression alterations might exist that will be common to and retraceable across different species and tissues. Apart from the pulsed expression of the superordinate core set of clock genes and related transcription factors in the brain hypothalamus [16], the aforementioned SCN-independent, self-sustained functionality of the CR is operating at the single-organ level, as it is well-described, e.g., for skin and liver [17]. Thus, evidence of SCN-independent CR regulation requires species with an evolutionary conserved decentralized CR, such as fish‚ and the inclusion of self-entrained organs.

In this study, we performed inter-species and inter-organ transcriptional analyses based on 329 RNA-Seq libraries to identify CR regulatory patterns in the course of aging. We identified differentially expressed genes (DEGs) by assessing the transcriptional profiles of mature, aged, and old-age individuals. We compared these results between *Homo sapiens* (*H. sapiens*), *Mus musculus* (*M. musculus*), *Danio rerio* (*D. rerio*), and *Nothobranchius furzeri* (*N. furzeri*), with the latter representing the family of extremely short-lived killifish, which is characterized by special adaption to ephemeral humidity of their habitats. The organs examined in this cross-sectional study comprised the brain, blood, liver, and skin.

We report 6 CR-related genes, i.e., *per2*, *dec2*, *cirp*, *klf10*, *nfil3*, and *dbp*, to exhibit conserved aging-related expression characteristics in up to 4 tissues of the species investigated. Additionally, we describe their role in tissue-inherent CR regulation and link them to age-associated processes and diseases. Our observations extend the understanding of the complex interplay between the circadian system and aging within vertebrates and provide a basis for further research on aging intervention strategies.

## MATERIALS AND METHODS

### Genomic and high-throughput transcriptomic data acquisition

**Figure 1:**
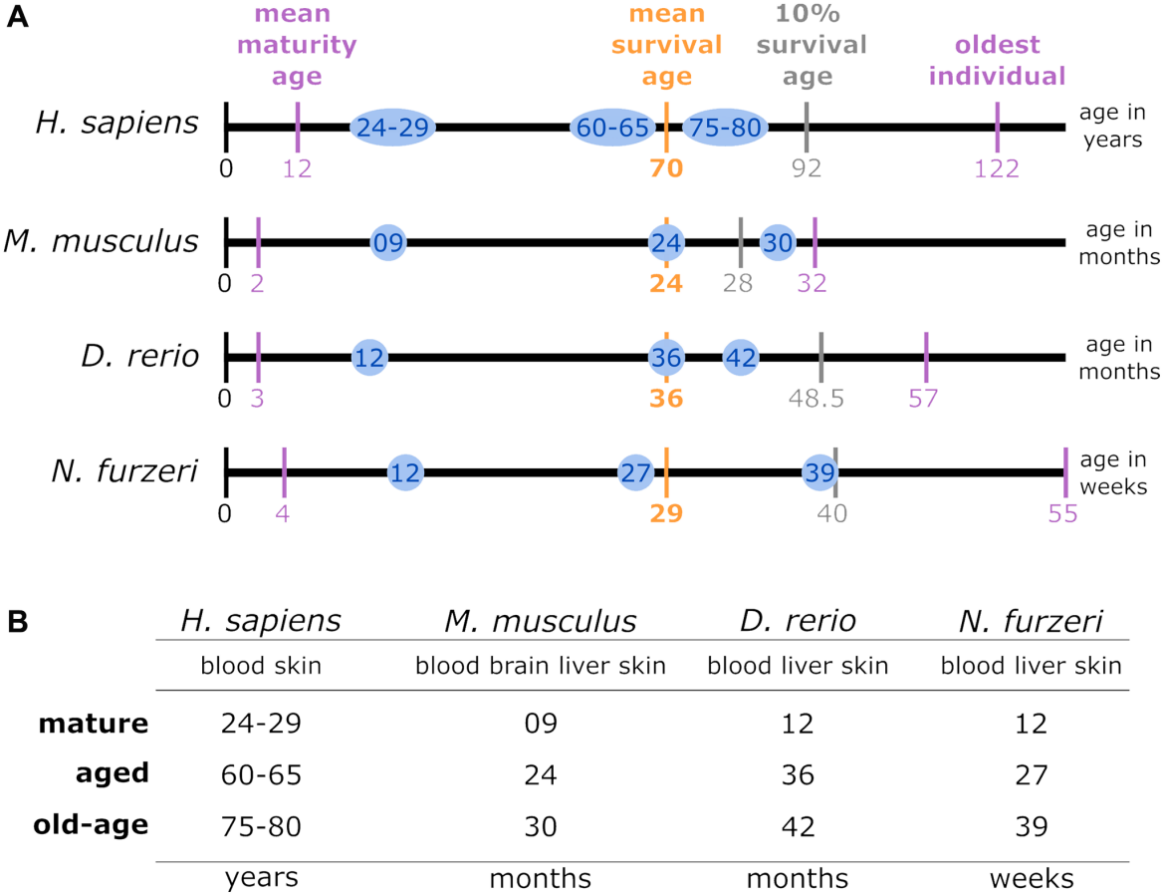
(**A**) Lifespan comparison: to align the species-specific chronological ages to biological age categories comparable between different species, the total individual’s lifetime, represented by the length of the lifetime axis, was subdivided by index stages. These stages were the biological stages corresponding to maturation, the mean survival age, the 10% survival rate, and the highest age reported for an individual belonging to the respective species. The time intervals between the resulting intersections were normalized linearly for the mean survival age. Thus, the sampling time points examined in this study for a specific species (blue circles) matched the biological age category in all the other species. (**B**) Data sampling scheme and categorization of the high-throughput transcriptomic data according to age parameters: for each of the 4 species of interest, up to 4 tissue types were sampled at mature, aged and old-age stages, from which 3 comparisons were deduced to identify DEGs during early aging (mature vs. aged), late aging (mature vs. old-age), and longevity (aged vs. old-age).

The genomes and annotations of *H. sapiens*, *M. musculus* and *D. rerio* used in this study were obtained from the Ensembl database (release version 92) [18]. For *N. furzeri*, the most recently published version of its genome assembly and the corresponding annotation were used [19, 20]. The RNA-Seq data analyzed within this study were initially published by Irizar et al. [21] and compiled by the JenAge Consortium (http://www.jenage.de/). The whole RNA-Seq dataset is available at NCBI’s Gene Expression Omnibus (*H. sapiens*: GSE75337, GSE103232; *M. musculus*: GSE75192, GSE78130; *D. rerio*: GSE74244 and *N. furzeri*: GSE52462, GSE66712). All human donors were classified into the following age groups: 24–29 years, 60–65 years, and 75–79 years (*n* = 14–15 for each category). For mice, the selected age groups were 9 months, 24 months, and 30 months, with blood and skin samples deriving from identical individuals. Liver and brain sampling was based on a separate subset of animals (blood, skin, liver, *n* = 5 for each; brain, *n* = 8). Tissues extracted from fish, i.e., *D. rerio* and *N. furzeri* comprised brain, liver, and skin (*n* = 5 for each) and were isolated at 12 months, 36 months, and 42 months and 12 weeks, 27 weeks, and 39 weeks of age, respectively (Figure 1). A total of 329 RNA-Seq libraries was examined. Within each species, the samples analyzed were collected at the same time of day. Further details regarding tissue sampling and extraction are available in the online supplement (https://osf.io/tkanf/).

### Lifespan normalization and transcriptomic data processing

The 4 investigated species differ with respect to their lifespans. To facilitate comparability, we normalized their lifespans to key biological stages: Time of maturity, average survival and extended survival reached by 10% of the individuals as well as the maximum reported age at death (see Figure 1A). According to these time scales, we defined 3 representative time categories comparable for each species – mature, aged, and old-age. Among these, we performed 3 different age comparisons denoted as early aging (mature vs. aged), late aging (mature vs. old-age), and longevity (aged vs. old-age) (see Figure 1B). Information on maturation and survival of the 4 species was underlying different sources: for *H. sapiens* [22–24]; for *M. musculus* [25–28]; for *D. rerio* [29–32]; and for *N. furzeri* [19, 33–35].

The RNA-Seq libraries were filtered and quality-trimmed with Prinseq (v0.20.3) [36], i.e., to achieve a minimum sequencing accuracy of 99.0%, all reads were truncated at both ends until a Phred quality score of 20 or more was reached. Thereby, reads below a length of 15nt or those comprising at least 3 ambiguous N bases were removed. Read qualities were monitored by FastQC (v0.11.3; https://www.bioinformatics.babraham.ac.uk/projects/fastqc/). TopHat2 (v2.1.1) [37] was used with default parameters to map the quality-trimmed RNA-Seq libraries to the corresponding reference genomes, thus, allowing spliced reads and single read mapping to multiple best-fitting locations. Featurecounts (v1.5.3) [38] was applied to perform read counting, with all reads being normalized to transcripts per million (TPM) [39] using the following formula:


$$TPM_{i}=\frac{c_{i}}{l_{i}}\times \left(\frac{1}{\sum_{j\in N}\frac{c_{j}}{l_{j}}}\right)\times 10^{6}$$

where *c_i_* indicates the raw read count of gene *i*; *l_i_* represents the cumulative exon length of gene *i*; *N* comprises the number of all genes in a given annotation. Genes with a TPM value ≤1 in each sample were considered not to be expressed and were excluded in all subsequent expression analyses. The Bioconductor DESeq2 (v1.10.0) package [40] was utilized to identify DEGs in the 3 age comparisons of every species and tissue studied. Benjamini and Hochberg’s False Discovery Rate (FDR) approach [41] was employed to adjust the calculated *p*-values for multiple testing. All genes with an adjusted *p*-value of less than 0.05 were assumed to be significantly differentially expressed. Details regarding DEG results, raw and normalized counts are accessible in the online supplement (https://osf.io/gtu2w/).

### Compilation of conserved CR genes and t-SNE dimension reduction

**Figure 2:**
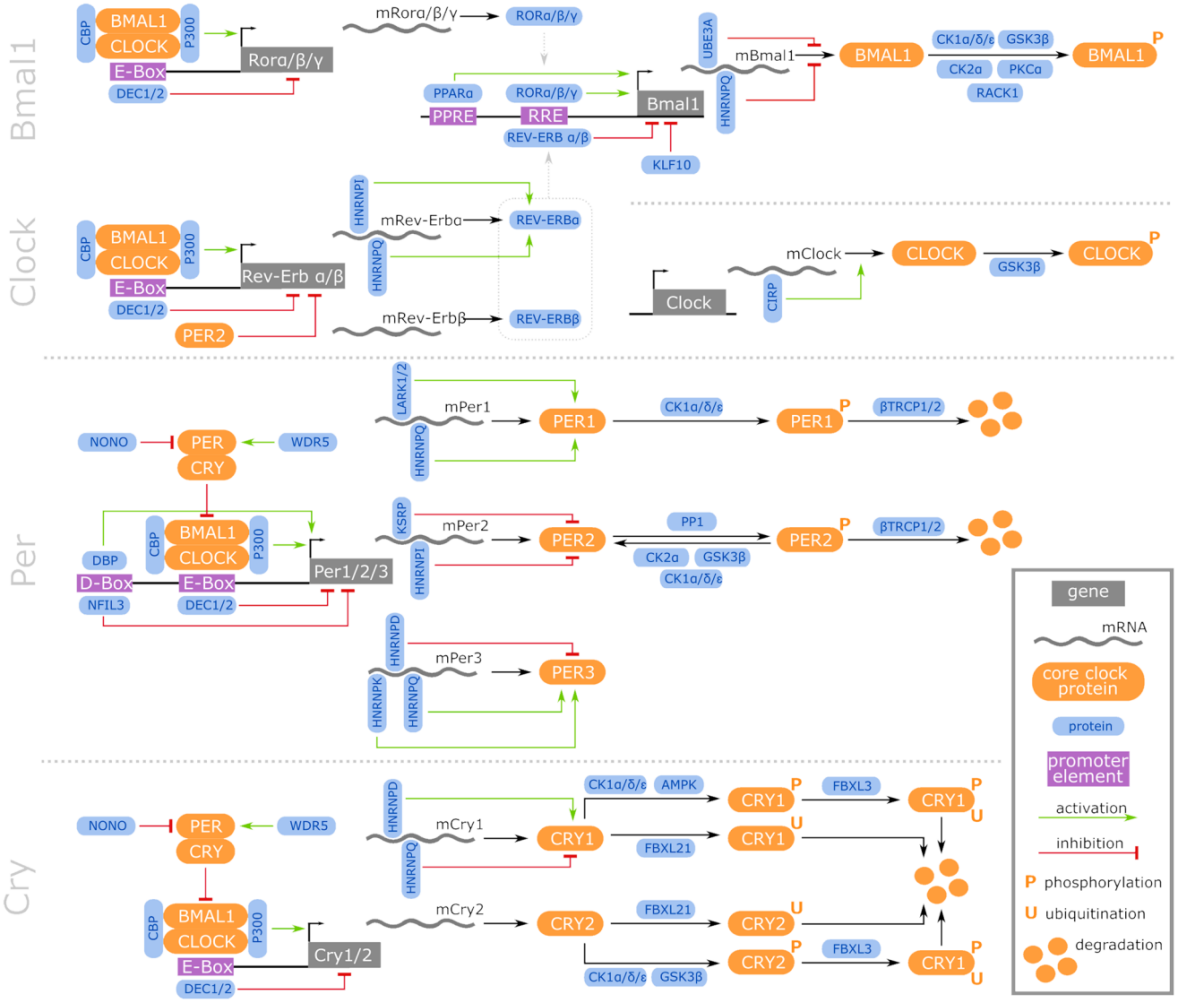
**Regulatory network of vertebrate core CR genes, including their transcriptional, post-transcriptional, and post-translational interaction partners that constitute activating and inhibitory feedback loops.** Further information is accessible in the online supplement https://osf.io/ctv2r/.

We focused on the core genes that establish the circadian cycle and their direct regulators. Based on a comprehensive literature-based data curation, a list of 46 CR-related genes common in all 4 investigated species was compiled (for details see online supplement https://osf.io/ctv2r/). These 46 CR-related genes were further categorized into 4 groups: 9 CR core genes (CRCGs), 11 transcriptional regulators of the CRCGs, 9 post-transcriptional regulators of the CRCGs, and 17 post-translational regulators of the CRCGs. Figure 2 illustrates the specific interplay between the CRCGs and their different regulators. In order to assess the expression similarities of the RNA-Seq data related to these 46 CR-related genes, in terms of the parameters species, organ and age, the t-distributed stochastic neighbor embedding (t-SNE) algorithm [42] was applied for dimensionality reduction (see online supplement https://osf.io/e7k9g/).

### Age-dependent gene expression variances

The coefficient of variation expressed as the ratio of the standard deviation and the mean of each CR-related gene was assessed to approximate the overall variance in gene expression among the reference time categories defined for the individual species and tissues. Variance differences among time categories were statistically assessed by two-tailed *t*-test and corrected for multiple testing by the Benjamini and Hochberg’s False Discovery Rate (FDR) approach [41].

### Data availability statement

The datasets analyzed for this study can be found in the NCBI’s Gene Expression Omnibus database (https://www.ncbi.nlm.nih.gov/geo/): (*Homo sapiens*: GSE75337, GSE103232; *Mus musculus*: GSE75192, GSE78130; *Danio rerio*: GSE74244 and *Nothobranchius furzeri*: GSE52462, GSE66712).

## RESULTS AND DISCUSSION

### Age-related effects on CR gene expression across species

Vertebrate CR has been well characterized in mice and zebrafish, but less in other species. Moreover, to the best of our knowledge, a comprehensive comparison of age-dependent CR regulation between phylogenetically distant species is not yet available. This study compares 4 tissues (brain, blood, liver, and skin) of 4 vertebrate species (the 2 mammals, *H. sapiens* and *M. musculus*, and the 2 fish, *D. rerio* and *N. furzeri*) at 3 different ages.

#### CR-related gene expression discriminates among species and tissues rather than age

First, we evaluated the homogeneity of the analyzed transcriptomic data. Thus, we applied the t-SNE dimensionality reduction algorithm on the investigated RNA-Seq libraries based on the 46 selected CR-related genes (see online supplement https://osf.io/e7k9g/). Thereby, samples clustered primarily at the species’ tissue level, i.e., no inter-species tissue cluster emerged. As the only exceptions, the 2 fish liver samples were merging and partially overlapped with the *N. furzeri* skin samples. Among all clusters, almost no age-specific segregation of either age category was discerned. This observation suggests that aging, from a global perspective, might have a relatively subtle impact on the global transcriptional level of CR-related genes, which seems to be a common finding shared by related studies addressing other target genes underlying the same dataset [43, 44]. However, a certain separation between the mature, aged, and old-age categories was observed for brain samples of *D. rerio*. For *N. furzeri*, only the old-age brain samples were distinct from the 2 younger time points. For *M. musculus*, no such separation was found. Additionally, the old-age skin samples of *N. furzeri* were distinguishable from the mature and aged samples. Human samples of either tissue were devoid of age-specific cluster formation.

#### General observations of age-related effects on the CR regulation across species

**Figure 3:**
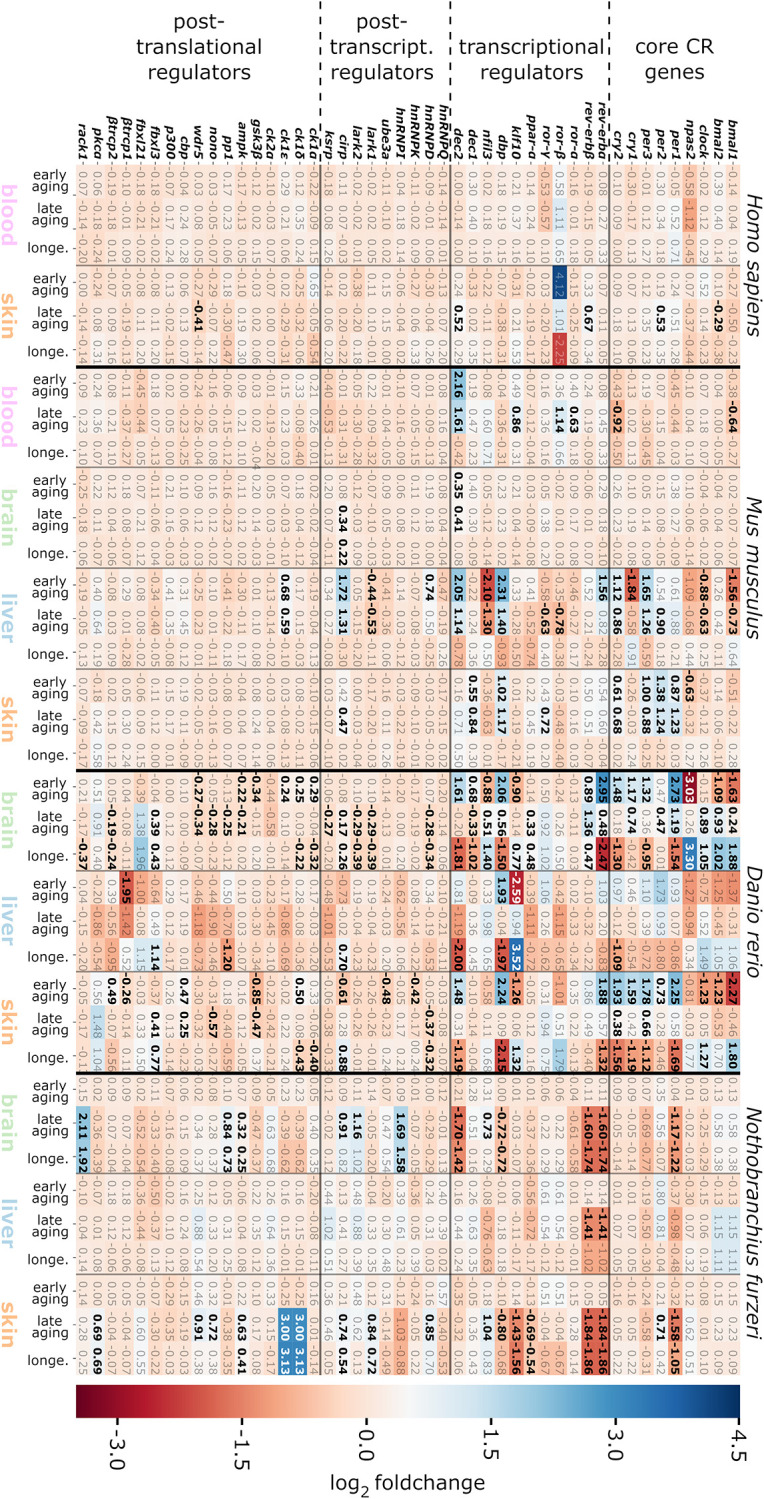
**Heatmap representing *log*_2_ fold changes of the CR-related genes for the species, tissues and age categories investigated.** DEG up-regulations in the course of aging are indicated by positive values displayed in blue, whereas down-regulations are shown by negative values in red. Significant gene expression alterations are highlighted in bold. The abbreviation longe. stands for the longevity age comparison (aged vs. old-age samples). For details, see the online supplement https://osf.io/9c3j4/.

However, at the single-gene level, we found 42 out of the 46 CR-related genes to be differentially expressed in an age-dependent manner in at least one species and one age comparison (see Figure 3). In total, out of these 42 DEGs, we identified 37 DEGs in *D. rerio*, 22 DEGs in *N. furzeri*, and 21 DEGs in *M. musculus*. In the human samples, we found 5 DEGs exclusively within the comparison between the mature and the old-age skin samples. Most of the DEGs identified belonged either to the CR core genes or to their direct transcriptional regulators.

A possible explanation for the low amount of DEGs identified in human samples might relate to the circumstance that the probands were not exposed to controlled environmental conditions and synchronization for day-night-cycle activity. Even though the human samples were collected during similar daytime, the variance in expression of individual genes was higher than within the other species. This higher intra-tissue gene expression variance in the human samples was also reflected by the broader sample distribution within the t-SNE projection. The age-related transcript changes represented by the 5 DEGs identified in the human samples, 2 of which belong to the CR core genes, might underestimate the impact of aging on human CR regulation.

Interestingly, represented by 16 DEGs, the most frequent transcript changes were found within liver samples of *M. musculus* aside from the DEGs manifesting in the skin, blood, and brain samples (9, 6, and 2 DEGs, respectively). Such observations are in accordance with the recent illustration that, at least in mouse, aging has a more profound impact on the liver than on other organs, in terms of gene expression levels, which might explain the pronounced number of DEGs within the mouse liver samples [44, 45]. This finding contrasted with the expression pattern discovered in both investigated fish species, where most of the DEGs arose from brain and skin specimens rather than from liver samples. A putative explanation might originate from the non-hierarchical and decentralized operation of the circadian system in fish [7]. Fish tissues are characterized by a direct light responsiveness and independent circadian pacemakers, which maintain the CR in different organs (for a recent and comprehensive review, see Steindal et al. [46]). Furthermore, since organs are differently affected by the sequela of aging, the peripheral representatives of the circadian system in fish might also respond individually with respect to their CR-associated gene expression patterns. In terms of biological requirements, each tissue might adapt individually to age-related influences on its organ-specific pacemakers as autonomous from a central pacemaker.

In mouse, representing mammals, *cirp* was the only DEG identified within the longevity comparison. Cirp is a post-transcriptional regulator of the CR core gene *clock* and of the crucial transcriptional CR regulator *dbp* [47]. In contrast, in *D. rerio* and *N. furzeri*, the comparison between aged and old-age samples, categorized as longevity, resulted in 25 DEGs and 16 DEGs, respectively, also including *cirp*. Such difference in CR regulation during longevity between mammals and fish might arise from a putatively earlier adaption to biological aging effects in the analyzed human and murine tissues or be due to the ability of fish to adjust their CR at higher ages.

In *N. furzeri*, the early aging comparison remained devoid of CR-related DEGs, which emerged only in comparison with the old-age samples. Therefore, the age-related changes in the CR regulation might have a positive effect on the longer lifespan of the old-age individuals. This explanation remains speculative, requiring further investigations of the CR in *N. furzeri*, similar to studies available in *D. rerio* [5, 48].

#### Conserved expression patterns of per2 and dec2 during aging

**Figure 4:**
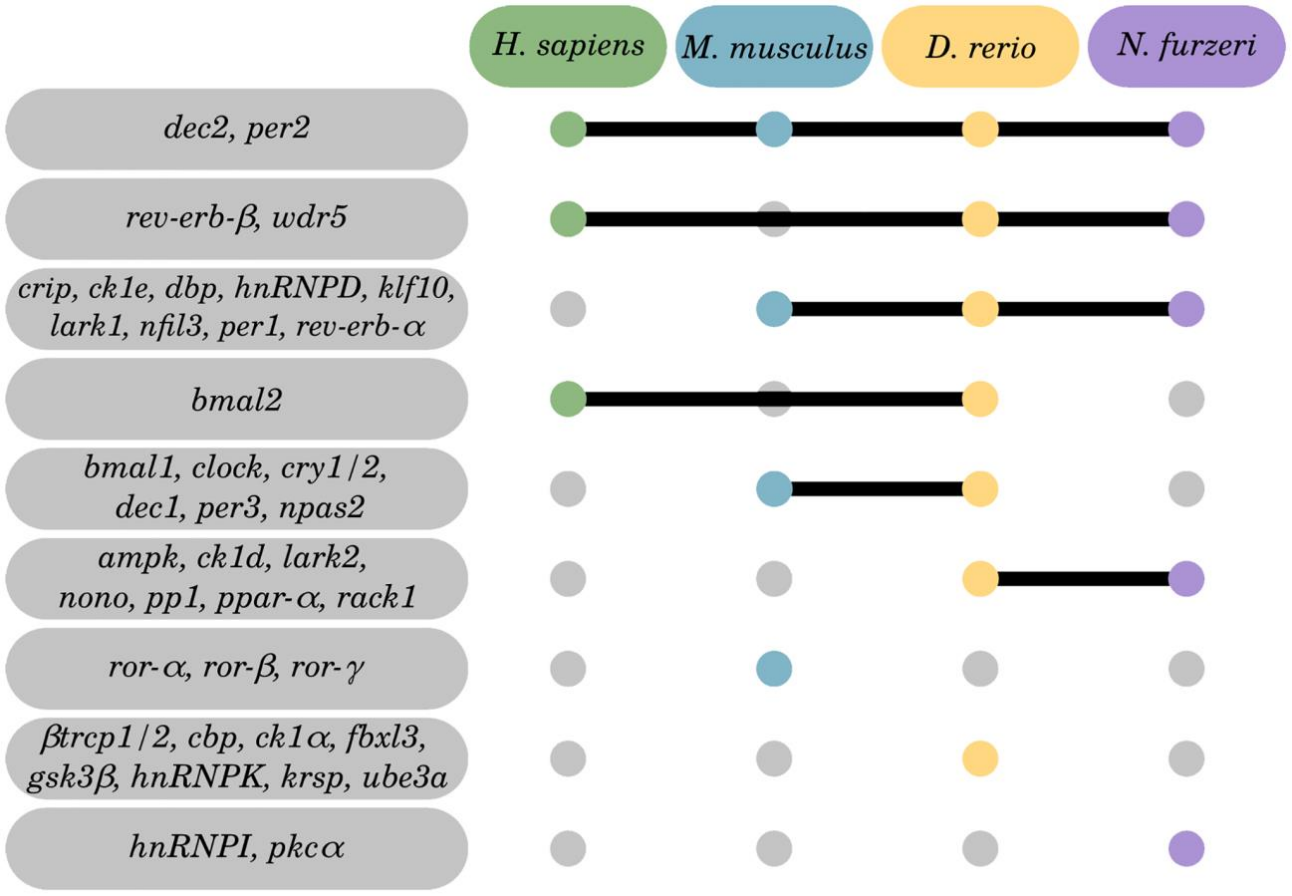
**Inter-species overlap of CR-related genes regulated by aging. Of the 42 identified DEGs, 28 were identified within 2 or more species, and 14 DEGs to be species-specific**. Tissue-specific Venn diagrams can be found in the online supplement: https://osf.io/3mgc6/.

Out of the 42 DEGs, 2 were shared in all 4 investigated species in at least one tissue, 11 in 3 species, 15 in 2 species, and 14 DEGs were identified to be species-specific (see Figure 4). Among them, the core circadian system regulator *per2* and its competitive transcriptional repressor *dec2* were differentially expressed during aging in all 4 investigated species.

##### Per2

*Per2* is a member of the *per* family and plays a crucial role in CR maintenance as one of its core regulators. As part of the negative branch of the CR regulatory feedback loop system, Per2 forms a heterodimer with Per1 and Cry1/2, thereby suppressing the Bmal1:Clock complex and inhibiting its own expression [49, 50]. Deregulation of *per2* is known to play a role in cancer risk and progression of several tumor types (for a brief review, see Wood et al. [51]). However, overexpression experiments correlated *per2* with a tumor-suppressing function [52–54]. In our data, we detected *per2* to be up-regulated with age in the skin of all 4 species analyzed, in the brain of *D. rerio* and in the liver of *M. musculus*. Such shared rise in its age-related expression suggests that prevention of tumor formation during aging might involve a conserved *per2* function. Considering its dual role, as a potential tumor suppressor and as a core regulator of the CR, age-related influences on *per2* regulation by other CR-related genes, like *bmal1* or *clock*, might potentiate its function in tumor suppression. Additionally, *per2* is described to stimulate autophagy levels in mammals [55]. Age-dependent decline in the efficiency of autophagocytic cascades is one of the main driving forces of aging [56] and restored autophagy was evidenced to prolong the lifespan of several organisms [57–59].

##### Dec2

Dec2 belongs to the basic helix-loop-helix family of transcription factors. It operates as a competitive transcriptional repressor of E-box-dependent *per* gene family transcription and thus plays a crucial role in executing the negative branch of the CR feedback loop as well as in CR downstream regulations [60, 61]. Across all age categories, we found *dec2* to be differentially expressed in each of the 4 species including the 4 tissues investigated. Whereas its expression was increased with age in both mammals, it was mainly decreased in fish, with the exceptions of an inconsistent regulation in *D. rerio* brain and skin samples. There, *dec2* displayed an increased expression in the early aging comparison, followed by a decrease in its transcript level in the old-age as compared to the aged individuals. Dec2 is involved in several other aging-related cellular processes, like tumor formation [62], hypoxia [63], apoptosis [64], and inflammation [65, 66]. Similar to *per2*, the expression of *dec2* is highly dependent on the interaction with other CR-related genes [61], making it prone for age-dependent deregulation of the CR. Moreover, *dec2* is established for its oscillating expression in the SCN. Here, our data provide indications that *dec2* expression and regulation is not restricted to the master CR pacemaker in the brain hypothalamus, but it is apparently also conserved in skin, liver, and blood. Whether *dec2* transcripts, similarly to *dbp*, belong to the targets of the post-transcriptional regulator Cirp, particularly in old tissue, requires further exploration [47].

### Tissue-specific gene expression changes over the course of aging

Although our set of target genes comprised 46 conserved CR-related regulators among the 4 investigated species, only a few of them showed a comparable expression pattern during aging, as discussed above. One reason is pulsed transcription of CR genes and the individual oscillatory responses to entrainment factors by different tissues [11, 67, 68]. Thus, concerning our analysis, we focused on age-related inter-species CR gene expression patterns underlying the same tissue rather than comparing several tissues from an individual species.

#### Brain

Brain-mediated CR regulation, beyond a key pacemaker role of the SCN, has so far not been extensively studied in the context of aging [69, 70]. Likewise, CR phase oscillations entrained by non-SCN brain tissue might be comparable to other non-SCN tissue as recently evidenced in the work by Yan and colleagues [68]. Also, deletion of CR key players in brain such as *bmal1/2* and *clock* in conjunction with *npas2* was identified to cause severe age-related astrogliosis [14]. By contrast, the deletion of *bmal1* alone lead both to structural and molecular changes [14].

In this study, brain-specific regulations of the CR-related transcriptome were characterized in mice and 2 fishes. As these brain analyses in mice and fish were based either on a hemisphere or a whole brain, respectively, our data are not specific for age-related changes eventuating in the hypothalamus. Age had almost no impact on the expression of CR genes in murine brain, independently of the age category addressed, suggesting a robust circadian system over mature lifetime. In contrast, CR-related genes were heterogeneously and strongly altered in both fish brains, thereby reaching *log*_2_ fold changes ranging from −3.03 to 3.30 in *D. rerio* and −1.74 to 2.11 in *N. furzeri* (see Figure 3). Thus, CR regulatory input from SCN-independent neuronal populations in fish brains (potentially in addition to the pineal complex and photoreceptors of the eye) might have stronger impact than in mammals or be subjected to pronounced age-related perturbations in fish. In an earlier study by Zhdanova et al. [5], the CR core genes *bmal1* and *per1* were described to be down-regulated in the pineal glands of aged zebrafish, but not *clock*. Here, we found an altered, diminished expression level of *clock* in the early aging comparison of the *D. rerio* brain samples as well. However, in the old-age *D. rerio* brain specimens the expression of these 3 CR core genes was stimulated both in comparison to mature and aged samples. The detection of *dec2* and *cirp* as the only DEGs that were altered both in the murine and fish brain samples might highlight their roles in the integration of peripheral CR oscillators, such as from liver and skin, into superordinate CR pulses.

##### Cirp

The highly conserved stress-inducible RNA-binding protein Cirp is known to directly interact and stabilize the mRNA transcripts of the CR core gene *clock*, enhancing its translation [47]. As *cirp* is also expressed in a broad tissue variety, the observed up-regulation of *cirp* in aging organs from mouse and both fishes might follow the biological purpose to counteract age-related CR alterations and stabilize the CR under increased cellular stress. Accordingly, *cirp* expression is responsive to hypoxia, UV radiation, body temperature, sleep patterns, metabolic cues, and DNA damaging stressors [71]. However, such role of *cirp* in the restitution of the CR robustness in the context of aging deserves further experimental proof.

Consistent with our evidence of a well-stabilized CR system in the brain, the resilience of the mammalian hypothalamus towards aging was recently demonstrated [72]. In this study by Eghlidi et al., underlying microarray and qRT-PCR techniques on young and old tissue specimens, the diurnal expression of SCN regulatory genes was unaffected by the age of the studied rhesus monkeys. Consequently, the authors concluded that CR misalignment in subordinate regulatory body systems might be the source of altered behavior in aged rhesus macaques. Moreover, our data indicate that, despite a decentralized CR regulation, CR core genes in fish are similarly conserved as in mammals, with SCN-paced hierarchical CR. Likewise, several fish orthologues of murine CR-interacting transcription factors represent targets of the CR core gene *bmal1* [15].

#### Blood

Although longitudinal CR studies are frequently performed with blood, the data from the cross-sectional approach used in this study argue for a rather weak age-related CR representation in this tissue. Likewise, no age-associated alterations of the CR-related transcriptome were detected in nucleated cells from human blood. In mice, only a few CR-related genes were identified to be differentially expressed, including representatives of the CR core gene category and some of their transcriptional regulators. The DEGs identified in murine blood displayed *log*_2_ fold changes between −0.92 and 2.16, with the strongest effects being found for *cry2* (*log*_2_ fold change: −0.92) and *dec2* (*log*_2_ fold change: 2.16). The Dec2 transcription factor sustains the negative arm of the circadian core feedback loop that suppresses the Bmal1: Clock protein dimer. Dec2 is involved in impaired sleep homeostasis and short sleep manifestation [73] and signals on pathways propagating tumor progression and growth [62], which, on the other hand, are altered by the aging process [74].

#### Liver

##### Kfl10

Liver represented the organ with the most frequent and most prominent CR-related gene expression alterations found in *M. musculus* and *D. rerio*, respectively, with the latter displaying *log*_2_ fold changes between −2.59 and 3.52 for the *klf10* orthologue. Klf10 is a crucial repressor of *bmal1* and was found down-regulated in the aged *D. rerio* liver samples compared to the mature and old-age samples, the 2 of which displayed similar expression levels. Reduced expression of *bmal1* is known to contribute to aging, e.g., through the association with a higher abundance of reactive oxygen species, thereby propagating cellular senescence, at least in mice [75, 76].

##### Nfil3

In both the early aging and the late aging comparison in mice, we found *nfil3* to be lower expressed (*log*_2_ fold changes of −2.1 and −1.3, respectively), suggesting that the amount of Nfil3 might be decreased in aged and, to a less extent, in old-age mice. Nfil3 binds to D-box elements residing in the CR core gene promoters, such as of the *per* family members *per1/2/3* (see Figure 2). Nfil3 negatively regulates the transcription of these genes by competing with PAR-domain basic leucine zipper (PAR bZip) transcription factors such as Dbp, Hlf, and Tef, in an anti-phasic oscillatory manner [77]. In accordance with the suppressor function of Nfil3 on the *per* family, its observed down-regulation coincided with an up-regulated expression of *per2* and *per3* in the same age comparisons. Similarly, we found *dbp* expression elevated in the corresponding age categories in mice, putatively entailing higher Dbp protein levels with age, again with a less pronounced regulation in the long-lived than in the aged individuals.

##### Dbp

Dbp is a D-box binding competitor of Nfil3, promoting the expression of *per* genes [78, 79]. The transcription of *dbp* itself is suppressed by *per* gene family members [78]. The deregulation of *per* genes has been associated to tumor progression and a poor cancer prognosis [54, 80]. In addition, some studies found that overexpressed *per1* and *per2* can act as tumor suppressors in a time-dependent manner in some malignancies [52–54]. In the *D. rerio* liver samples, the expression of *dbp* was, similarly as observed for mouse samples, increased in the early aging comparison with a *log*_2_ fold change of 1.93. Its expression was decreased in the longevity comparison with an equally strong *log*_2_ fold change of −1.97, i.e., levels of *dbp* transcripts were only elevated within the aged but not within the old-age individuals as compared to the young mature individuals. Dbp acts as a transcription factor of *per* family genes, and also controls the expression of many metabolic and detoxifying enzymes within the liver [81]. A reduced expression of *dbp*, along with other PAR bZip transcription factors, is correlated with epilepsy and early aging symptoms in mice [81, 82].

#### Skin

CR-related gene alterations in skin were represented across all 4 species, thereby showing *log*_2_ fold changes between −2.27 (for *bmal1* in *D. rerio*) and 3.13 (for *ck1δ* and *ck1ε* in *N. furzeri*). The most prominent expression changes were detected in *D. rerio*, engaging significant changes in 24 out of the 46 target CR-related genes. A similar pattern was found in the skin of *N. furzeri* featuring significant changes in 17 out of the 46 target CR-related genes. Regarding human and murine samples, the number of CR-related DEGs was less abundant (5 out of 46 in human skin; 9 out of 46 in mouse skin). Overall, 4 out of 46 genes were shared as DEGs within the skin samples of at least 3 species, including genes encoding for Per1 and Per2 of the core clock regulators, as well as Dbp and the RNA binding protein Cirp (Figure 4).

For *cirp*, we observed a higher expression either within the aged or old-age skin samples of *M. musculus* and both fish, similar to its expression pattern in the brain samples. As aforementioned, Cirp interacts with the Per members and with Dbp by stabilizing their transcript and protein levels. Dbp belongs to the PAR bZip family of transcription factors, which can activate the Per1 promoter in cooperation with Clock/Bmal1, while Clock/Bmal1 stimulates the transcription of Dbp. By contrast, Dbp transcription is suppressed by Per members [78]. Thus, these data support the assumption that the post-transcriptional regulator Cirp may play a key conserved role in adjusting CR oscillations under aging conditions. Thereby, it may influence core clock members and associated transcriptional modulators such as Per1, Per2, and Dbp, thus enabling a reciprocal consolidation of CR circuits. In how far Cirp exerts such an assumed CR stabilizing function under aging conditions, particularly in the skin, has to be further analyzed.

When addressing the early aging category in *D. rerio* skin samples, the DEGs found to be up-regulated comprised representatives primarily of the negative branch of CR core regulatory genes. In contrast, transcript levels of core regulator genes of the positive branch were down-regulated. Strikingly, this regulation was reverted to the contrary pattern within the longevity comparison. Collectively, this finding implies that expression of CR core genes, except for *per3* and *cry2*, was only altered within the aged individuals but remained stable within the old-age compared to the younger mature individuals. Such observation suggests that the exceptionally long-lived animals might have peculiar molecular measures to stabilize their CR. A similar pattern was observed in the *D. rerio* brain samples. However, the altered CR core gene expression levels were increased in the old-age individuals when compared to the mature individuals. A similar complementary regulation between the early aging and the longevity comparisons was discerned for the transcriptional regulators *klf10*, *dbp*, and *dec2*.

By contrast, the killifish *N. furzeri* showed no aging-related reversion in the expression of CR-related genes in none of the organs analyzed. The respective CR-related genes reflected either a consistent increase or decrease in expression with aging. Thus, concerning the execution of chronobiological studies, zebrafish might be favorable over *N. furzeri* to monitor temporal CR alterations.

In murine skin samples, we detected an augmented expression of all CR core genes of the negative CR feedback loop branch during aging, except for *cry1*, the expression of which remained stable. Additionally, the transcriptional regulators *ror-γ*, *dbp*, *dec1*, and the post-transcriptional regulator *cirp* were also higher expressed with age.

### Differences in the expression stability of aged and long-lived individuals

**Figure 5:**
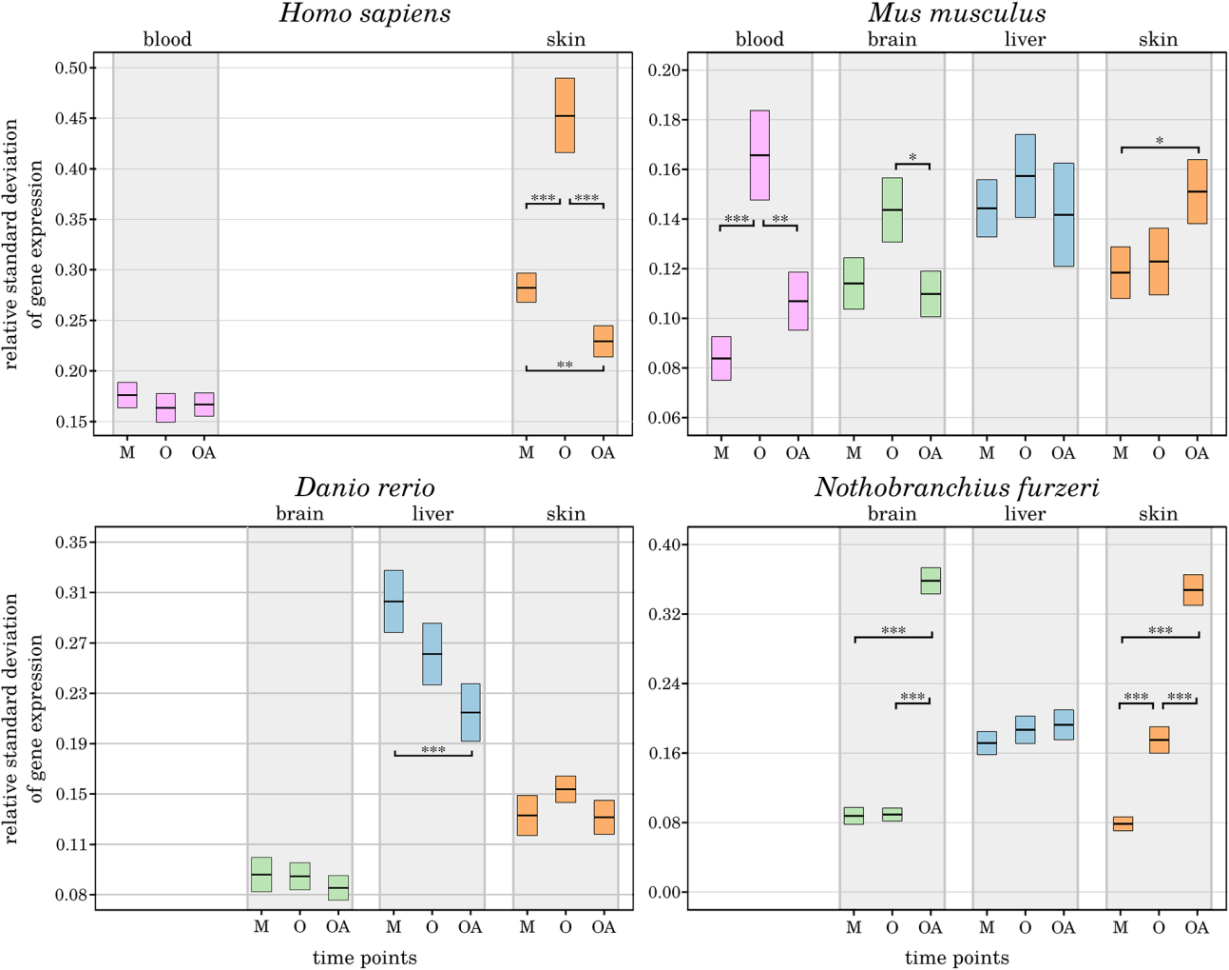
**Age-dependent alterations in the robustness of CR-related gene expression.** For the 3 age comparisons, different tissues, and species, the measured variance in the expression of CR-related genes is displayed as change in standard deviation. The extrema of the box plots represent the respective 2.5% percentiles. Age groups (M – mature, A – aged, OA – old-age) are compared for their statistical significance: ^*^p-value ≤ 0.01, ^**^p-value ≤ 0.001, ^***^p-value ≤ 0.0001. Details can be found in the online supplement: https://osf.io/g9uqz/.

Aging is suspected to be responsible for disrupting the circadian system’s regulation on both the master clock regulator level and the peripheral oscillators [4, 6]. Moreover, aging might be associated with an altered scatter of gene expression variances and serve as an indirect biostatistical indicator of organ and tissue aging [43]. Therefore, average changes in gene expression variances were specified for the 3 age comparisons delineated and applied as measure for the robustness of CR-related gene expression (Figure 5). In our previous study, we found genetic marker genes of senescence and inflammation to exhibit lower standard deviations in the old-age samples, indicating a more robust expression of those genes in the long-lived individuals [43]. However, for the CR-related genes a corresponding pattern was not discriminated. In 7 out of 16 species-related tissues within 4 tissue types, we detected no or only minor differences in the relative standard deviations between the age groups. The remaining tissues displayed heterogeneous changes between the respective ages, without the perception of a consistent pattern.

While human and mouse samples shared an overall increased variance in CR-related gene expression in the aged individuals, the long-lived individuals exhibited an unchanged or even lower expression variance than the younger mature individuals. The only exceptions were the human blood samples, without substantial age-related impact on the expression variances. The mouse skin samples showed an increase in gene expression variance towards the old-age time point.

In *D. rerio*, the inter-group variability in gene expression was constantly decreasing with age selectively in liver samples. Exclusively in *N. furzeri*, the expression variances reflected a consistent pattern of an increased relative standard deviation towards old-age, pointing to a less robust expression of the CR. This observation supports our finding that DEGs were primarily arising from the old-age category in *N. furzeri*. Longitudinal studies are required to characterize the robustness of CR-related gene expression patterns within the different tissues and species.

### Cross-over mapping with established CR regulomes

The primary shortcoming of our current study is rooted in the single time point-sampling of tissues and species included and the deficiency of a DEG expression normalization to a zeitgeber time (ZT) and inter-species alignment to rhythmic CR gene expression. To validate, in consideration of this caveat, the assignment of our underlying data to a chronobiological context, the set of CR-related DEGs exhibiting age-related alterations were aligned to input data [83] as well as enriched output data of a newly established, refined high-confidence network of CR-regulated genes in humans, called NCRG. This reference network was retrieved by a bioinformatical meta-analysis of multi-modular high-throughput CR-related data [84].

**Figure 6:**
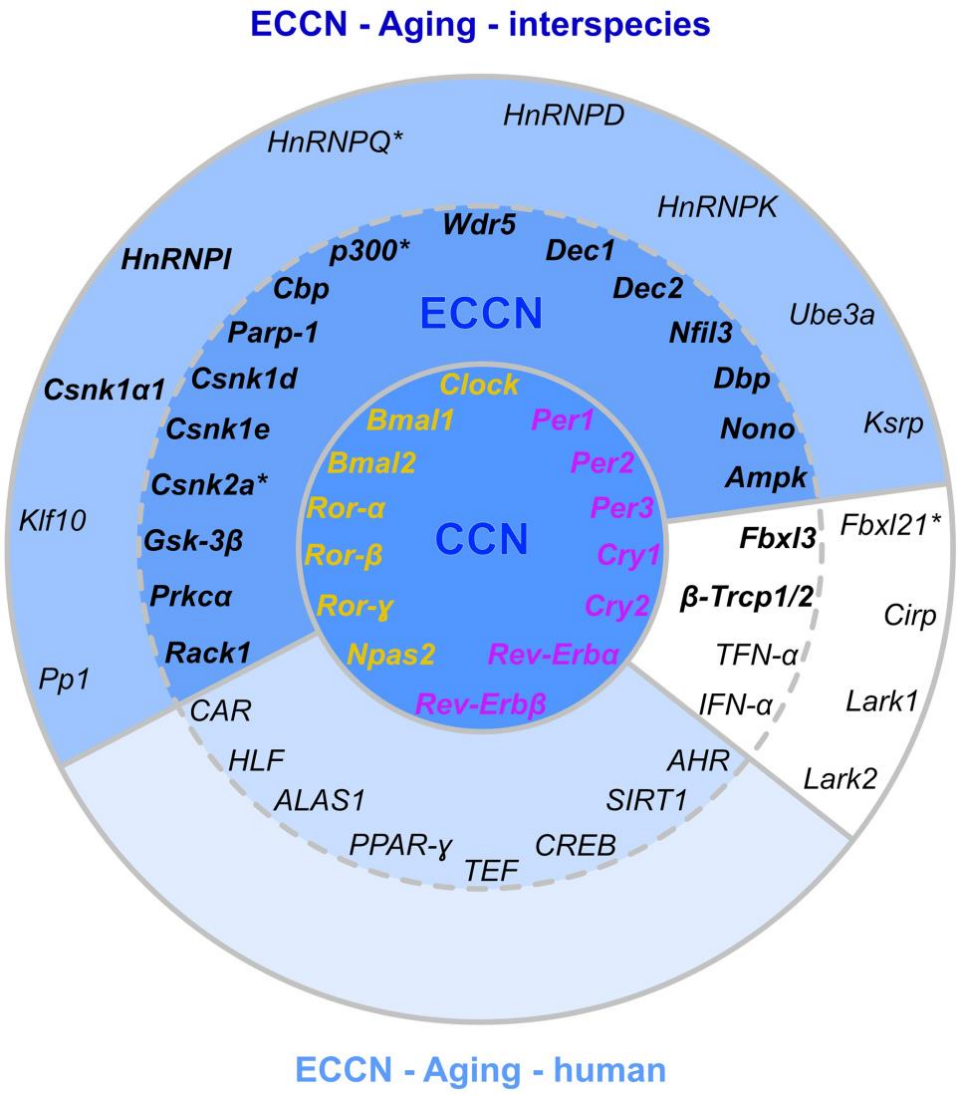
**Matching of our CR-related DEGs evidenced to be regulated with aging with a curated human CR network.** The core clock network (CCN; inner ring) is comprised of a positive (orange) and negative (purple) branch of core clock regulators and has been expanded to an extended core clock network (ECCN; middle ring) as determined by chronobiological meta-analyses in humans [84]. The majority of CR-related genes identified in the present study overlaps (represented in bold) with the human CCN (100% overlap) and ECCN (65.5% overlap). The ECCN was complemented by 13 CR-related genes defined by our aging-related interspecies approach (outer ring), two of which overlapped with newly designated CR genes (bold in outer ring [84]). By mapping to a curated biomedical aging database [101], 85% of the presented interspecies (dark blue background) or human (light blue background) CR-related genes were identified to be aging-associated. The remaining factors (white background) have not yet been annotated to aging but might represent new candidates. ^*^, curated CR-related genes without age-related differential expression within the interspecies comparison.

The underlying input inter-connectome of a core clock (CCN) and an extended core clock network (ECCN) implementing update connections according to PubMed-available data comprised 43 CR-related elements displaying more than 200 regulatory interdependencies [83, 84]. First, when mapping our 46 CR-related genes (Figure 3), 42 out of which proved to be differentially expressed during the aging process, we identified that all of the 14 CR genes nominated to be engaged in the CCN in humans [84] were represented in the category of core clock regulators and immediate transcriptional regulators in our inter-species study. This 100% overlap covered both the positive and negative branches of the circadian feedback loop (Figure 6). Second, as for the ECCN, 19 out of 29 (65.5%) CR-related elements designated to belong to the human ECCN [84] overlapped with the DEGs nominated as CCN interaction partners in our inter-species approach (Figure 6). Third, among the 118 genes, newly nominated by Lehmann and colleagues to be potential ECCN targets and thus becoming part of their innovative, most comprehensive NCRG in humans, we found 2 of them, i.e., *Csnk1α1* and *HnRNPI*, to overlap with our DEGs output. As the reference network established by Lehmann and colleagues is not tissue-specific, we could not match for inter-tissue peculiarities.

Considering such correspondence, this large-scale curated human CR-regulatory network might be well complemented by the human homologs of the 13 additional genes we *de novo* assigned to the CR regulome in our non-synchronized but inter-species and tissue-specific approach, 11 of which represented DEGs with a significant expression modulation by aging (Figure 6). These additional genes encompass one transcriptional and numerous post-transcriptional and post-translational regulators. In support of such notion, only 19 out of the 118 genes newly assigned to the expanded NCRG network suggested by Lehmann and colleagues showed circadian expression patterns in mice [86]. Thus, mutual overlap and uniqueness of DEGs in the inter-species comparison might broaden our evolutionary view on candidate genes relevant in hierarchical CR regulation, development of CR-related disorders in humans, and chronotherapy.

Among these additional 11 CR-related DEGs, the structurally conserved gene encoding for the Klf10 zinc finger transcription factor of the Krüppel-like family was found engaged in the class of transcriptional regulators. Klf10 functions as an effector of the TGF-β signaling pathway. It operates as a repressor of cell proliferation and inducer of apoptosis and thus is assumed to play a role in cancer growth and progression. Moreover, Klf10 is indicated to propagate cardiovascular diseases, diabetes, and cancer by activating pro-inflammatory tissue responses [85].

The DEGs assigned to the category of post-transcriptional regulators included several representatives of the HnRNP class of RNA interacting factors (*HnRNP D, Q, I, K*; *HnRNPA18*/*Cirp*), a large, conserved family of RNA binding proteins [86]. HnRNPs assemble on newly generated heterogeneous nuclear transcripts (hnRNA) to control their conversion into mature mRNAs. Along with this, they are involved in (pre-) mRNA processing, trafficking, and splicing. By transcriptome diversification, alternative splicing holds an established role in the adaptive stress response activated during aging. Accordingly, deregulation of the spliceosome has been identified as a hallmark of aging, as it is associated with aging phenotypes and the expression of aging biomarkers in humans and age-related disorders [87, 88]. In terms of transcript affinity, many of the HnRNPs have oncogenes as direct targets as well as factors crucial in age-related neurodegenerative disorders, such as *TERT/TERC, KRAS, BRCA,* and *APP* mRNAs [88].

Similarly, the gene encoding the RNA-binding K-homology splicing regulatory protein Ksrp identified among these 11 CR-related DEGs, has a key function in regulating mRNA stability of genes involved in immune and inflammatory responses. As a member of the AU-rich element-binding proteins (ARE-BP), it controls gene expression by targeted decay of mRNAs [89]. Ksrp prevents excess and harmful cytokine activation as a dominant target, e.g., after infection, in chronic neurodegenerative disorders, and cancer [90]. Repression of Ksrp target genes has been discussed to serve as a conserved anti-inflammatory mechanism across different species, including humans, mice, and rats, and across different tissues and cell types [91].

Further, ubiquitin E3 ligases, such as the product translated from *Ube3A* mRNA we detected among these DEGs, have been associated with several age-related neurodegenerative disorders. *Ube3A* is involved in genomic imprinting of experience-dependent ocular plasticity during development, and loss-of-function models exhibit synaptic rigidity typical of the aged and neurodegenerative brain [92, 93]. Ube3A protein levels have been shown to decline by 50–80% with aging in the mammalian cortex, as illustrated for humans, macaque monkeys, and cats, suggesting that this loss in Ube3A contributes to impaired cortical functionality and synaptic plasticity at higher ages [94].

Likewise, we found *lark1* and *lark2* to be among these 11 DEGs emerging in the inter-species approach. Products of the RNA binding *lark* genes have recently been identified to stabilize G-quadruplex structures in vertebrates and invertebrates in a conserved process [95] thus enhancing the transcription of target genes. As a primary target, Lark activates the expression of *per1*, thereby fundamentally regulating the length of CR periods [96]. Notably, *lark* transcript levels are not subjected to rhythmic pulses, whereas protein levels oscillate synchronously with Per1 in mice [96]. Such characteristics make *lark* an interesting novel candidate in understanding altered CR periodicity observed with aging.

Isoforms of the CsnK family (e.g., α, β, γ, δ, ε) and their splice variants have a vital role in the phosphorylation of molecules regulatory in transcription, translation, receptor-coupled signaling, apoptosis, cell cycle control, DNA repair, and chronobiology [97]. Further, they operate in multiple oncogenic signaling pathways [97]. Notably, the regulated CsnK2α is discovered to phosphorylate Sirtuin 6 [98], which also operates as a longevity factor in mice.

Moreover, the post-translational CR regulator Pp1 has a function in the control of memory-related miRNAs. The activity of the Pp1 protein phosphatase is altered with aging, suggesting an implication in the manifestation of age-related cognitive impairments and dementia syndromes associated with progressive neurodegenerative disorders [99].

Further, the post-translational *fbxl* gene family, including the nominated *fbxl21* gene, is involved in cellular processes critical in aging and age-related pathologies such as cell cycle control and DNA damage response. The encoded F-box proteins represent E3 ubiquitin ligases that recruit their substrates and target them for degradation via interaction with specific motifs, or degrons, primed by post-translational modifications [100]. Deregulations are best characterized for cancer entities but suggest participation in pathologies implicating genomic stress conditions, including the aging process.

For additional support of the biomedical interpretation that our output DEGs are relevant for the aging process, we correlated our 46 CR-related genes with the entirety of genes currently annotated for a relevant function in aging. For this purpose, we utilized a newly established integrated database collecting and updating unprecedented multi-scale and in-depth knowledge on the biology of aging [101]. Notably, this publicly accessible biomedical database curates multi-omics information on the process of aging across different model organisms and species (https://bigd.big.ac.cn/aging/index). When applying the RNA-Seq database within the catalog of available data collections, we show that 39 out of 42 DEGs and 46 annotated genes summarized in our heatmap (85%) are linked to the aging process. The high overlap of our results with pre-established bioinformatic CR pipelines and age-related functional -omic scale databases, as illustrated in Figure 6, supports the strength of our data despite a single time point study design.

## CONCLUSION

Multiple efforts have aimed at mapping physiological and pathological processes to the circadian system either in individual [12, 102] or multiple organs [11] of a single species or in selected organs underlying a pathophysiological stressor [103]. However, the role of highly conserved CR regulation in physiological aging over time and across multiple species has not yet been addressed. Here, we used RNA-Seq data to profile the regulation of CR-related genes of 4 different species in a cross-sectional study in individuals ranging from young mature to old-age categories. We compared changes of the CR transcriptome in different organs within these species, including brain, blood, liver, and skin. In summary, our results show that modulations in CR-related gene transcription throughout aging are a conserved trait that is traceable across evolutionarily diverse species, ranging from humans to mice and fish. Thereby, we identified 2 CR-related genes, i.e.*, dec2* and *per2*, to be altered in all 4 species primarily in early aging and late aging rather than longevity, and 4 genes (*cirp*, *klf10*, *nfil3*, and *dbp*) with conserved aging-related expression patterns in several organs and species.

In contrast to the analysis of CR-related mRNAs from different tissues, as studied, e.g., in zebrafish larvae [15], we realized an inter-organ comparison. In complementation to the work by Yan et al. (2008), who surveyed 14 tissues exclusively from mammalian species (i.e., human, monkey, and mouse) from a tissue gene expression atlas [68], we expanded the analyses of inter-organ relationships to 4 evolutionarily separated species and specified the regulation of CR-related genes in the context of aging. In comparison, the study by Yan and colleagues compiled microarray data from several previous studies and extracted 41 common CR genes from a total of 9,995 genes that oscillated in at least 8 out of 14 tissues in mice [68].

In our study, among the 4 species investigated, *H. sapiens* showed less regulation in terms of both the number and absolute expression changes of DEGs irrespective of the reduced availability of only 2 out of 4 tissue locations analyzed. If DEGs were identified, they belonged, except for *wdr5*, exclusively to the core clock genes or the category of immediate core clock transcriptional regulators. *M. musculus* exhibited a similar hierarchical pattern of age-dependent CR regulation in terms of the 4 categories of gene regulators defined, concluding that primarily core CR genes and their transcriptional regulators were among the DEGs identified. In murine tissues, this pattern was best reflected by liver and skin. Such preponderance in the executive level of operating genes and their expression alterations was also present in *D. rerio*, with the strongest age-associated regulations found for core CR genes and transcriptional regulators. However, in contrast to mouse and human, *D. rerio* also showed broad age-related changes in the CR transcriptome at the level of post-transcriptional and post-translational regulators. In general, *D. rerio* exhibited the most versatile CR gene modulations concerning DEG numbers, *log*_2_ assessed gene expression alterations, tissues being involved in CR regulation, and gene regulatory categories covered. Thereby, the most prominent changes were attributable to brain tissue followed by skin. Unexpectedly, the short-lived killifish *N. furzeri*, which serves as an established aging model, was devoid of a corresponding CR regulatory pattern. Strikingly, we found almost no regulation of the core clock genes except for *per* family members. In general, the number of age-related CR genes in *N. furzeri* was much lower than in *D. rerio* and consistently represented by the gene regulatory categories downstream of the core CR genes. Accordingly, in contrast to *D. rerio*, the strongest DEG regulation manifested within post-translational regulators instead of core CR genes. Unlike *M. musculus*, both fishes showed a more complex involvement of brain and skin instead of liver in this aging-related CR pattern. In conclusion, *D. rerio* appeared to be best equipped to respond to aging-related CR-alterations in terms of transcriptome adaptions among the 4 species analyzed.

In general, we identified alterations in the CR regulation as an early characteristic of aging, in *M. musculus* and *D. rerio*, which emerged at or persisted into late aging in human, mouse, and fish, and further extended into longevity in *D. rerio* and *N. furzeri*. Though manifesting already during early aging, the inter-tissue and inter-species profile of DEGs engaged in CR regulation appeared broadest during late aging but less diverse at the old-age time points and best represented in their complexity by brain and skin. Thus, apart from a master role of the brain SCN in CR control, our data suggest an essential contribution of the skin to the transmission of chronobiological cues, as we detected a considerable amount of DEGs in the skin across all 4 species, possibly explained by the direct light exposure of this organ. Notably, in the 2 fishes, DEG patterns in the skin were comparable to that in brain tissue. In mice, age-related changes in CR-regulatory transcript levels were evident in the skin but not represented by brain tissue. These findings suggest that even in species relying on a primarily hierarchical CR orchestration, as found in mice and humans, peripheral subordinate organs such as the skin might compete with hypothalamic brain structures and contribute to CR-related gene expression alterations during aging.

In support, expression of repressive core clock *per2* in the skin was increased during aging in all 4 species investigated. Age-related alterations of CR-related gene expression in light-sensitive skin suggest multi-level changes in the stimulus-dependent responsiveness of the CR regulatory system. Importantly, the regulation of *per2* is mediated by complex feedback loops directly involving the *cis*-regulatory D-box enhancer element, which is recognized by PAR bZip and related transcription factors such as Hlf, Tef, CR activating Dbp, and repressive Nfil3. As aforementioned, apart from *per2* itself, *dbp* and *nfil3* were found differentially expressed by age in mice and the 2 fish species, although D-box regulation differs fundamentally between mammals and fish (see online supplement https://osf.io/zc8d3/). Whereas in mammals, stimulation of D-box-activating transcription factors is indirectly regulated by interlocking feedback loops encompassing the CR output targets Bmal1 and Clock, D-box-binding transcription factors, such as Tef, are directly inducible by light in fish, conveying light-entrained regulation of clock genes [104]. Along with visible light, also UV light and ROS levels are engaged in D-box-driven, light-responsive gene regulation in fish but not mammals, targeting clock and DNA repair genes such as *ddb2* relevant in nucleotide excision repair (NER) and other stress response pathways [105, 106, 107]. Similarly, ROS levels are critically elevated during aging. In conclusion, we identified 3 CR genes, i.e., *per2*, *dbp*, and *nfil3*, as being differentially regulated with aging, which are involved in direct light-induced gene expression in fish while representing clock output elements in mice and humans. The strong light responsiveness of D-box elements in fish might explain the pronounced expression alterations detected for *dbp* and *nfil3* in the skin of fish, while in the mouse samples, D-box regulation was dominant in the liver.

Apart, the diametrical expression of *dbp* and *nfil3* illustrated in our heatmap for the murine liver is supported by other studies. Likewise, Ueda and colleagues identified an anti-phasic expression of these 2 transcription factors in frame of transcriptional circuit analyses established from *in vivo* liver and vector-transduced cellular models [79].

Additionally, we identified 6 genes in several tissues of at least 3 out of the 4 species investigated with an apparently conserved age-associated regulation. These genes act at each of the regulation levels established for the CR (core gene, transcriptionally, post-transcriptionally, and post-translationally) and have known biological roles outside the CR. Many of these functions are related to aging-associated diseases or driving factors, such as autophagy, hypoxia, apoptosis regulation, and inflammation. Thus, these genes might represent a conserved link between the circadian system and aging. However, since preliminary due to the non-synchronized cross-sectional approach, our study deserves future replication in an additional dataset based on a longitudinal study design, including tissue synchronizations across the species of interest and the consideration of anatomic sub-regions when brain tissue is analyzed.

In how far CR regulation is causal to or a consequence of the aging process still remains to be explored. This work extends recent studies and yields a comprehensive new dataset linking CR factors to physiological aging across evolutionarily distinct species. It will support to dissect the alterations in chronobiology associated to latent or manifest disease conditions from CR characteristics in healthy aging and contribute to the identification of interventions that can improve the well-being of the elderly and extend a healthy lifespan.

## Supplementary Materials

Supplementary Data
