# Supplementary material for: Age-dependent expression changes of circadian system-related genes reveal a potentially conserved link to aging
Source: Aging (Albany NY). 2021 Dec 19;13(24):25694–716. doi: 10.18632/aging.203788 (PMC8751596; doi:10.18632/aging.203788)
Supplement: Supplementary Data [file aging-13-203788-s001.pdf]

SUPPLEMENTARY DATA

All supplementary Data can be found at the Open Science Framework: https://osf.io/aydjt/.
